# Supplementary material for: ‘Sustainable Choices’ - An intervention to promote climate change mitigation behaviours: Study protocol for a randomized controlled trial
Source: PLoS One. 2026 Mar 31;21(3):e0346303. doi: 10.1371/journal.pone.0346303 (PMC13038011; doi:10.1371/journal.pone.0346303)
Supplement: Attachment — Submitted filename: report_PONE_D_25_45042.pdf [file pone.0346303.s001.pdf]

Report for manuscript PONE-D-25-45042: " 'Sustainable Choices' - An Intervention to Promote Climate Change Mitigation Behaviours: Study protocol for a randomized controlled trial"

Summary

The manuscript proposes a randomised controlled trial protocol to evaluate the "Sustainable Choices" online intervention to promote climate change mitigation behaviours. The protocol is well motivated, and its overall discussion is satisfying. Nevertheless, the statistical methodology section is not exhaustive, and some gaps must be addressed before it can be considered appropriate.

1 Major comments

1. **Primary outcome:** The authors propose the use of a modified version of the GEB instrument (GEB-CCMB), which is not validated and whose psychometric properties will be assessed at baseline. This choice naturally raises concerns, given that a poorly specified scale would invalidate latent-score estimation and, thus, the subsequent analysis proposed by the authors. In addition, the authors' assumptions about the latent trait(s) they aim to measure are not explicit. In particular, it is unclear whether they assume a unidimensional trait (CCMB) or a multidimensional space (energy, mobility, food, consumption, finance, and advocacy). Specifically, I am referring to the sentence *"In addition to overall scores, domain-specific θ-scores will be computed for each behavioural category (energy, mobility, food, consumption, finance, and advocacy)"*. If the unidimensionality assumption holds, subdomain scores might not provide any additional information beyond the general unidimensional score (CCMB), as all subdomains will be highly correlated (literally overlapping with the CCMB). If the multi-dimensionality assumption holds, the general unidimensional score (CCMB) will not be reliable; instead, the authors will have to treat behavioural dimensions as correlated latent traits. Finally, the mixed-format nature of the responses to the proposed scale (stage of adoption, frequency, counts) might also make it difficult for the unidimen-

sionality assumption to hold. Appropriate references in this regard can help the authors justify their choice.

2. **Sample size:** The proposed sample size of 680 individuals seems reasonable to detect differences in means between the two arms, but the exact number given is not fully transparent. In particular, it is not clear which test statistics the authors are using to base their calculations. Furthermore, it might be helpful to provide a reference for the considered "allowance of 30% data loss". In addition, a sensitivity analysis of the sample size to different values of g would also benefit the overall robustness of the proposal.

3. **Missing data:** The authors suggest using the `missForest` package to handle eventual missing values. However, there is no discussion of the types of missing values they expect to observe. In IRT model estimation via marginal maximum likelihood, missing values do not need to be imputed if the MAR assumption holds. Imputation, in this case, might be more harmful than helpful. Therefore, the questions that follow naturally are: which variables do the authors expect to observe missing values (questionnaire responses, respondents' characteristics, ...) and under which assumption (MCAR, MAR, ...)?

4. **Statistical models:**

   - It is not clear to the reader how the IRT model(s) will be estimated. In particular, my concern is whether the model will be fitted separately to the two arms or jointly to the full sample. In both cases, there are some concerns that should be addressed:

     – **Separate estimation for time and arms:** Given the 5-point Likert scale described in the manuscript, a single fit on a sample of data collected from the authors' GEB-CCMB scale would involve the estimation of at least 150 parameters (30x4 thresholds + 30 discrimination parameters + latent correlations in the multi-dimensional case). The size of a single arm will not be adequate to estimate such a model. Furthermore, without formally showing that item parameters are invariant between the two arms, it will not be possible to compare latent traits, as they will be scored on different metrics.

     – **Joint estimation across time/arms:** This choice is more appropriate to obtain θ-scores comparable across arms, however it is not clear how time dependency (repeated observations per indivudual) will be accounted.

   - Net of the unclearness regarding the employement of IRT models, the current use of θ-scores as dependent variable in a subsequent regression analysis is a weakness on its own. The authors propose to use the latent traits estimated via the IRT model (for primary

and secondary outcomes) as dependent variables in a subsequent regression phase. While computationally convenient and conceptually straightforward, such a strategy has a major caveat: it neglects the variability of latent trait estimates. Therefore, the proposed strategy could lead to an underestimation of the variability of the estimated regression coefficients and, thus, invalidate the final hypothesis testing phase. From my perspective, the authors have three possible choices: 1. The optimal choice is to jointly model the IRT and regression phases by considering, for example, a structural equation modelling approach; 2. Stick to the two-step estimation but switch the analysis to a Bayesian approach, to draw multiple samples from the posterior distribution of each θ-score. Using multiple posterior samples per observation as the dependent variable in the regression analysis will mitigate the issue, but is still sub-optimal as a joint analysis should be preferred; 3. Stick to their proposed procedure, but at least run some sensitivity analysis based on the estimated standard errors of the θ-scores, and clearly acknowledge that θ-estimates have heteroskedastic variability (typically with higher variance at the extremes of the dimension) and, thus, regression standard errors and inference will be affected.

- Similarly to the IRT models, it is not clear how data correlation due to repeated observations on the same individual will be handled in the regression model.
