# Supplementary material for: ‘Sustainable Choices’ - An intervention to promote climate change mitigation behaviours: Study protocol for a randomized controlled trial
Source: PLoS One. 2026 Mar 31;21(3):e0346303. doi: 10.1371/journal.pone.0346303 (PMC13038011; doi:10.1371/journal.pone.0346303)
Supplement: Attachment — Submitted filename: Response to reviewers.docx [file pone.0346303.s002.docx]

# Response to the reviewers

Dear reviewers,

Thank you for thorough and constructive feedback on our study protocol manuscript. Below you find point-by-point responses. Changes in the manuscript are visibly marked in a “tracked changes” version, resubmitted with this revision.

Best regards,

Dr. Fabian Lenhard, on behalf of all coauthors

Department of Clinical Neuroscience

Karolinska Institutet

Stockholm, Sweden

# Reviewer 1

***Comment 1:***
I agree that using a sample of voluntary individuals, probably with a high motivation for behavioural change, is a selection bias that may compromise the generalizability of the results. The authors may suggest possible solutions for future further research in order to avoid this problem, while very common in this type of studies.

***Response:*** We agree with this important point and think that future evaluations of the intervention should involve representative population samples. We have added a sentence regarding this in the Limitations section.

***Comment 2:***

In the conclusion section (307) there is a minor mistake (I think "the integration artificial intelligence" was "the integration of artificial intelligence").

***Response:*** This mistake is corrected now.

***Comment 3:***
Please check again the text to be sure to use British or American spelling consistently.

***Response:*** The manuscript has been spell-checked in British English.

# Reviewer 2

The manuscript proposes a randomised controlled trial protocol to evaluate the “Sustainable Choices” online intervention to promote climate change mitigation behaviours. The protocol is well motivated, and its overall discussion is satisfying. Nevertheless, the statistical methodology section is not exhaustive, and some gaps must be addressed before it can be considered appropriate.

***Comment 1:***

Primary outcome: The authors propose the use of a modified version of the GEB instrument (GEB-CCMB), which is not validated and whose psychometric properties will be assessed at baseline. This choice naturally raises concerns, given that a poorly specified scale would invalidate latent-score estimation and, thus, the subsequent analysis proposed by the authors. In addition, the authors’ assumptions about the latent trait(s) they aim to measure are not explicit. In particular, it is unclear whether they assume a unidimensional trait (CCMB) or a multidimensional space (energy, mobility, food, consumption, finance, and advocacy). Specifically, I am referring to the sentence “In addition to overall scores, domain- specific θ-scores will be computed for each behavioural category (energy, mobility, food, consumption, finance, and advocacy)”. If the unidimensionality assumption holds, subdomain scores might not provide any additional information beyond the general unidimensional score (CCMB), as all subdomains will be highly correlated (literally overlapping with the CCMB). If the multi-dimensionality assumption holds, the general uni- dimensional score (CCMB) will not be reliable; instead, the authors will have to treat behavioural dimensions as correlated latent traits. Finally, the mixed-format nature of the responses to the proposed scale (stage of adoption, frequency, counts) might also make it difficult for the unidimensionality assumption to hold. Appropriate references in this regard can help the authors justify their choice.

***Response:***

We thank the reviewer for this important comment.

First, we clarify that the “GEB-CCMB” referred to in the protocol has been published as a preprint under the name “Climate Mitigation Behaviour Scale” (CLIMBS). The earlier terminology reflected its developmental origin within the General Ecological Behaviour framework. Since the original submission (August 2025), the full psychometric evaluation of CLIMBS has been completed, submitted as an independent manuscript, and made publicly available as a preprint [1]. We have now harmonised terminology throughout the manuscript and refer consistently to the instrument as CLIMBS.

The development paper demonstrates that a bifactor graded response model provided the best representation of the data, with a strong general factor and four orthogonal domain factors: Mobility, Food, Consumption, and Engagement. The final 23-item solution showed good global fit (RMSEA = .045, CFI = .92, TLI = .91), satisfactory total reliability (ω_total = .83), and substantial general-factor saturation (ω_H = .69).

Thus, CLIMBS is best characterised as a hierarchical construct with a dominant general mitigation propensity, alongside interpretable but partly general-saturated domains. In line with these findings, the RCT protocol has been revised to specify that:

- The primary outcome is the general CLIMBS latent factor (EAP score), reflecting overall climate mitigation behaviour. Domain scores (Mobility, Food, Consumption, Engagement) are treated as secondary/exploratory outcomes, given their coexistence with a strong general factor.
- Regarding the mixed response formats (frequency, counts, stage of adoption), the development study modelled all items using a graded response IRT framework appropriate for ordered categorical data. The bifactor structure demonstrated good fit despite heterogeneous response anchors, indicating that the shared latent structure is defensible.
- The availability of the dedicated psychometric manuscript strengthens the methodological foundation of the present protocol. The RCT therefore does not rely on an unexamined instrument, but on a scale with a documented bifactor structure, established reliability, and evidence of external validity.

We hope this clarification addresses the reviewer’s concerns and have revised the Primary outcome section accordingly.

***Comment 2:***

The proposed sample size of 680 individuals seems reasonable to detect differences in means between the two arms, but the exact number given is not fully transparent. In particular, it is not clear which test statistics the authors are using to base their calculations. Furthermore, it might be helpful to provide a reference for the considered “allowance of 30% data loss”. In addition, a sensitivity analysis of the sample size to different values of g would also benefit the overall robustness of the proposal.

***Response:*** We thank the reviewer for this comment and have clarified the sample size calculation in the revised manuscript.

The calculation is based on detecting a between-group difference in the primary outcome (general CLIMBS latent score) using a two-sided test of mean differences (α = .05, power = 90%). Assuming a standardized mean difference of g = 0.3 (small-to-moderate effect), approximately 260 participants per arm are required. The total target sample (N ≈ 680) includes a 30% inflation to account for anticipated attrition, which is consistent with commonly reported dropout rates in behavioural and digital intervention trials [2,3].

We have also added a brief sensitivity analysis showing the detectable effect sizes across a plausible range (g = 0.25–0.40).

***Comment 3:***

Missing data: The authors suggest using the missForest package to handle eventual missing values. However, there is no discussion of the types of missing values they expect to observe. In IRT model estimation via marginal maximum likelihood, missing values do not need to be imputed if the MAR assumption holds. Imputation, in this case, might be more harmful than helpful. Therefore, the questions that follow nat- urally are: which variables do the authors expect to observe missing val- ues (questionnaire responses, respondents’ characteristics, ...) and under which assumption (MCAR, MAR, ...)?

**Response:** We thank the reviewer for this important observation and agree that imputation of item-level data in IRT models is generally unnecessary under a marginal maximum likelihood framework.

We have therefore removed the planned use of missForest. For CLIMBS item responses, missing data will be handled directly within the IRT estimation procedure, which accommodates incomplete response patterns under the assumption of missing at random (MAR).

We have added a brief description of how patterns and extent of missing data will be examined (e.g., proportion, distribution, and associations with observed variables). If missingness in covariates or outcome data exceeds trivial levels, appropriate regression-based methods consistent with the assumed missingness mechanism will be applied.

***Comment 4:***

Statistical models: It is not clear to the reader how the IRT model(s) will be estimated. In particular, my concern is whether the model will be fitted separately to the two arms or jointly to the full sample. In both cases, there are some concerns that should be addressed:

– Separate estimation for time and arms: Given the 5-point Likert scale described in the manuscript, a single fit on a sample of data collected from the authors’ GEB-CCMB scale would involve the estimation of at least 150 parameters (30x4 thresh- olds + 30 discrimination parameters + latent correlations in the multi-dimensional case). The size of a single arm will not be adequate to estimate such a model. Furthermore, without formally showing that item parameters are invariant between the two arms, it will not be possible to compare latent traits, as they will be scored on different metrics.

– Joint estimation across time/arms: This choice is more appropriate to obtain θ-scores comparable across arms, however it is not clear how time dependency (repeated observations per indivudual) will be accounted.

Net of the unclearness regarding the employement of IRT models, the current use of θ-scores as dependent variable in a subsequent regression analysis is a weakness on its own. The authors propose to use the latent traits estimated via the IRT model (for primary and secondary outcomes) as dependent variables in a subsequent regression phase. While computationally convenient and conceptually straightforward, such a strategy has a major caveat: it neglects the variability of latent trait estimates. Therefore, the proposed strategy could lead to an underestimation of the variability of the estimated regression coefficients and, thus, invalidate the final hypothesis test- ing phase. From my perspective, the authors have three possible choices: 1. The optimal choice is to jointly model the IRT and re- gression phases by considering, for example, a structural equation modelling approach; 2. Stick to the two-step estimation but switch the analysis to a Bayesian approach, to draw multiple samples from the posterior distribution of each θ-score. Using multiple posterior samples per observation as the dependent variable in the regression analysis will mitigate the issue, but is still sub-optimal as a joint analysis should be preferred; 3. Stick to their proposed procedure, but at least run some sensitivity analysis based on the estimated standard errors of the θ-scores, and clearly acknowledge that θ-estimates have heteroskedastic variability (typically with higher variance at the ex- tremes of the dimension) and, thus, regression standard errors and inference will be affected. Similarly to the IRT models, it is not clear how data correlation due to repeated observations on the same individual will be handled in the regression model.

***Response:***

We thank the reviewer for these important methodological points and have clarified the analysis plan accordingly.

*Regarding IRT estimation and comparability across arms/time***:** Item parameters will be estimated jointly using a single bifactor graded response model to ensure that all scores are placed on a common metric across arms and assessment waves.

*Regarding repeated measures (time dependency).* Repeated observations within individuals will be handled in the outcome analysis using mixed-effects models (e.g., participant-level random intercepts) or equivalent marginal approaches, ensuring that within-person correlation is appropriately accounted for.

*Regarding use of θ-scores in regression.* The primary analyses will use the general CLIMBS score as the dependent variable in regression models. Given the satisfactory reliability of the general factor (ω_total ≈ .83), attenuation due to measurement error is expected to be limited. As a robustness check, parallel analyses using the unit-weighted total score will be conducted.

**References**

1.	Lenhard F, Fernández de la Cruz L, Axelsson K, Nerini FF, Mataix-Cols D. The Climate Mitigation Behaviour Scale (CLIMBS) – Development and psychometric evaluation. PsyArXiv; 2026. Available: https://osf.io/preprints/psyarxiv/76mdg_v1/

2.	Eysenbach G. The Law of Attrition. Journal of Medical Internet Research. 2005;7: e402. doi:10.2196/jmir.7.1.e11

3.	Melville KM, Casey LM, Kavanagh DJ. Dropout from Internet-based treatment for psychological disorders. Br J Clin Psychol. 2010;49: 455–471. doi:10.1348/014466509X472138
